# The outcome of combination of low dose oral prednisolone with propranolol for the treatment of infantile haemangioma

**DOI:** 10.12669/pjms.321.6390

**Published:** 2016

**Authors:** Muhammad Zulfiqar Anjum, Khawaja Haroon Khurshid Pasha, Syed Husnain Abbas, Muhammad Zubair

**Affiliations:** 1 Dr. Muhammad Zulfiqar Anjum, Assistant Professor of Paediatric Surgery, Department of Paediatric and Neonatal Surgery, Quaid-i-Azam Medical College, Bahawalpur, Pakistan; 2 Dr. Khawaja Haroon Khurshid Pasha, Principal / Head of Department, Department of Paediatric and Neonatal Surgery, Quaid-i-Azam Medical College, Bahawalpur, Pakistan; 3 Dr. Syed Husnain Abbas, Post Graduate Registrar, Department of Paediatric and Neonatal Surgery, Quaid-i-Azam Medical College, Bahawalpur, Pakistan; 4 Dr. Muhammad Zubair, Professor of Paediatric Surgery, Department of Paediatric and Neonatal Surgery, Quaid-i-Azam Medical College, Bahawalpur, Pakistan

**Keywords:** Infantile hemangioma, Management, Combination of low dose oral Prednisolone with oral propranolol, Effectiveness

## Abstract

**Objective:**

To determine the outcome of combination of low dose oral Prednisolone with oral propranolol for the treatment of infantile hemangioma.

**Methods:**

The patients fulfilling the inclusion criteria were registered through outpatient department. Diagnosis was confirmed clinically and on Color Doppler ultrasonography (CD). All the patients were given oral prednisolone in a dose of 1mg/kg/day and propranolol in a dose of 0.5mg/kg/day twice a day and increased up to 1.5mg/kg/day BID within three days with close monitoring of heart rate, blood pressure and blood glucose as inpatient. Treatment was given for three months then titered down for two weeks before cessation of treatment. The follow-up of patients were performed at 7^th^ day, at 1^st^ month and finally at 3^rd^ month. Treatment compliance was checked during each visit along with outcome parameters i.e. response which was excellent, good, moderate slight improvement and no effect. All the information’s were collected. Data was analyzed by using SPSS version 10.

**Results:**

Out of total 73 patients, 36.99% (n=27) were one year of age, 32.88% (n=24) were two years of age and 30.13% (n=22) were three years of age, mean± SD: 1.96±0.54 years, 53.42% (n=39) were male and 46.58% (n=34) were females, frequency of response of the treatment was recorded as 56.16% (n=41) had excellent, 23.29% (n=17) had good, 15.07% (n=11) had moderate response, 4.11% (n=3) had slight improvement and 1.37% (n=1) had no effect while frequency of acceptable outcome revealed as acceptable in 79.45% (n=58) while 20.55% (n=15) had not acceptable outcome

**Conclusion:**

The frequency of acceptable outcome of combination of low dose oral Prednisolone with oral propranolol for the treatment of infantile hemangioma is higher.

## INTRODUCTION

Infantile hemangiomas (IHs) are the most common vascular tumours of infancy, occurring in 5% to 10% of infants.^1^ Mostly infantile hemangiomas are small, but they can be large, disfiguring lesions with serious complications. Hemangiomas might also involve the orbit, airway, or visceral organs, such as the liver, brain intestinal tract, or lungs.^2^ They generally become evident within first few days of life and are characterized by an initial phase of rapid endothelial cell proliferation during the first year of life followed by a phase of slow involution.^3^

Until recently’ the mainstay of treatment of IHs has been corticosteroids in various forms, including topical, intralesional, and oral formulations, with the most common being oral prednisolone. The response rate was found to be 84%, with the greatest response occurring in children treated in the early proliferative phase of the lesion.^4^ The standard treatment regimen is 2 to 4 mg/kg of oral prednisone or prednisolone daily.^2^ Other treatment modalities for complicated IH include interferon alfa-2a, imiquimod, vincristine, cyclophosphamide, pulsed-dye laser, and most recently, propranolol.^5^,^6^

Propranolol is a nonselective beta-blocker that has been in use for around four decades by cardiologists, endocrinologists, pediatricians, and psychiatrists. Its safety profile is well establishedwhen given to appropriate patients.^7^ Propranolol has been associated with adverse events such as bradycardia, hypotension, hypoglycemia and bronchospasm.^8^,^9^

Oral prednisolone and propranolol have shown excellent results individually for the treatment of problematic IH. However, the combination of the two drugs in lower doses may also be used for the treatment of IH to avoid the complications associated with high doses of both drugs. One such study showed 95% results when treated with prednisolone and propranolol in combination.^10^

This mode of treatment for infantile hemangiomas is very safe and cost effective. The studies in this regard are lacking, few studies are available internationally, but no national/local study is available. Therefore, this will help us making local policies for patients and help in reducing the morbidity.

## METHODS

It is a descriptive case series study conducted at the Department of Neonatal & Pediatric Surgery and Plastic Surgery Bahawal Victoria Hospital, Bahawalpur from Jan 2012 to July 2012. Total of 73 cases were included. The patients of infantile haemangioma of both sexes with age ranging from 0-3 years of size more than 10mm over body surface (head’neck’chest and arms) were included.

Patients having known cardiac problems, bronchial asthma, previously treated either medically or surgically and Patients with lack of consent and incomplete follow up and hemangiomas involving internal organs/body cavities and those who need urgent treatment due to impingement on vital structures were excluded.

Seventy three cases fulfilling inclusion criteria were registered through Outpatient Departments of Neonatal & Pediatric surgery, and plastic surgery, Bahawal Victoria Hospital, Bahawalpur. Diagnosis were confirmed by consultant pediatric Surgeon (assistant professor and above having 5 years’ experience) clinically and by Color Doppler ultrasonography (clustered dilated, tortuous blood vessels with Sluggish blood flow) by consultant radiologist (assistant professor and above having 5 years’ experience). Demographic history including age (in years) and sex (male or female) were taken. Approval from the hospital ethical committee was taken. Written informed consent was taken from the parents/guardians of the patients after discussing the risks and benefits of the drugs.

All the patients received treatment with oral prednisolone in a dose of 1mg/kg/day and propranolol in a dose of 0.5 mg/kg/day twice a day (BID) and increase up to 1.5mg/kg/day BID within three days with close monitoring of heart rate, blood pressure and blood glucose as an inpatient. Treatment was given for three months to all patients then titer down for two weeks before cessation of treatment. In case of early response i.e. before three month, drugs were titered down for two weeks before cessation.

The follow up of the patients were performed at 7^th^ day after initiation of treatment, then 1^st^ month and finally at 3^rd^ month. Heart rate, blood pressure, random blood sugar and treatment compliance was checked during each visit along with outcome parameter i.e. response which was categorized as excellent, good, moderate, slight improvement or no effect taking good to excellent response as acceptable outcome by measuring the size of IHs in terms of maximum dimensions in millimeters by using 100 mm horizontal scale on photograph taking by using 12 mega pixel digital camera. All the information was collected on a specially designed proforma.

All the collected data was entered into SPSS version 10 and analyzed. The qualitative data like demographics (sex; male or female), response (excellent, >75%), good (50-75%), moderate (25-50%), slight improvement (<25%) or no effect (0%) and acceptable outcome was described as frequency distribution. Good to excellent response (more than 50% decrease in size) was considered as acceptable outcome.

Quantitative data like age (in years) and size of IH (in millimeters) was presented as means and standard deviations. The stratification was done for effect modifiers i.e., age, sex, and type of hemangioma. Post stratification Chi-square test was applied. P<0.05 was taken as significant.

## RESULTS

Seventy three patients fulfilling the inclusion/exclusion criteria were enrolled to determine the outcome of combination of low dose oral Prednisolone with oral propranolol for the treatment of infantile hemangioma.

Age distribution of the patients showed that 36.99% (n=27) had one year of age, 32.88% (n=24) had two years of age and 30.13% (n=22) had three years of age, mean±SD: 1.96±0.54 years. (Table-I)Gender distribution of the patients showed that 53.42% (n=39) were male and 46.58% (n=34) were females. (Table-II)

**Table-I:** Stratification for frequency of acceptable outcome with regards to age and age distribution(n=73).

Age(in years)	Acceptable outcome		P value
	Yes	No	
0-1	21(77.78%)	06(22.22%)	0.940
>1-2	19(79.17%)	5(20.83%)	
>2-3	18(81.82%)	4(18.18%)	

Total	=SUM(ABOVE) 58(79.45%)	15(20.55%)	

P value=0.00

**Table-II:** Stratification for frequency of acceptable outcome with regards to gender and gender distribution(n=73).

Gender	Acceptable Outcome		P value

	Yes	No	
Male	30(76.92%)	09(23.08%)	0.567
Female	28(82.35%)	06(17.65%)	

Total	58(79.45%	15(20.55%)	

Size of IH was recorded. Frequency of response of the treatment was recorded as 56.16% (n=41) had excellent, 23.29% (n=17) had good, 15.07% (n=11) had moderate response, 4.11% (n=3) had slight improvement and 1.37% (n=1) had no effect. Frequency of acceptable outcome revealed as acceptable in 79.45% (n=58) while 20.55% (n=15) had not acceptable outcome.

Stratification for frequency of acceptable outcome with regards to age was recorded, out of 58 cases, 68.97% (n=40) were between 1-2 years, 31.03% (n=18) had 3 years of age, p value was calculated as 0.00. (Table-I)

Stratification for frequency of acceptable outcome with regards to gender showed that out of 73 cases, 53.42% (n=39) were male, 46.58% were females, p value was calculated as 0.50. (Table-III)

**Table-III:** Stratification for frequency of acceptable outcome with regarding type of haemangioma (n =73).

Type of Haemangioma	Acceptable Outcome		P value

	Yes	No	
Localised	56(96.55%)	2(3.45&)	0.000
Segmental	2(13.33%)	13(86.67%)	

Patients were also observed for any short term complications, coushingoid fascies (n=1) gastric irritation (n=1) and also for long term complications. Short term complications were mild and transient and no long term complication occurred.

## DISCUSSION

Infantile hemangiomas (IH) are the most common infantile tumor, with a frequency of 4-10%.^11^ Recently there has been an interest in propranolol and other beta-blockers in the treatment of IH. Propranolol may be more effective and safer than previously established therapies, and may be an alternative when more widely accepted treatments for IH have failed. Initial studies suggest that it may also be used as a first-line therapy.

A previous study,^12^ compared the clinical effectiveness of oral propranolol with that of oral prednisone in the treatment of infantile hemangiomas (IH) and concluded that Propranolol appears superior to oral prednisone in inducing more-rapid and greater clinical improvement in this study.

This mode of treatment for infantile hemangiomas is very safe and cost effective. The studies in this regard are lacking, few studies are available internationally, but no national study is available. Therefore, the results of the study may help us by making local policies for patients and help in reducing the morbidity.

The findings of the study are in agreement with Buckmiller LM who reported that ninety seven percent (97%) of patients showed improvement in the quality of their treated in hemangiomas with propranolol therapy while 60% of the patients showed a final excellent response with more than 75% reduction in the size of the lesion (P<0.001), 20% showed a good response with more than 50% decrease in size of the IH, 16.6% showed a moderate response with less than 50% reduction in size and only one patient 3.3% showed no response to treatment.^13^

Another study by Koaya ACA showed 95% results when treated with prednisolone and propranolol in combination.^10^ some-other trials may be done to confirm these findings.

### Limitations of the study

As very few studies are available nationally/internationally and these results may be considered as primary.

## CONCLUSION

We conclude that the frequency of acceptable outcome of combination of low dose oral Prednisolone with oral propranolol for the treatment of infantile hemangioma is higher but more studies are required to confirm these findings as no local study is available for comparison.

## References

[1] Kilcline C, Frieden IJ (2008). Infantile hemangiomas: how common are they?A systematic review of the medical literature. Pediatr Dermatol.

[2] Kliegman RM, Behrman RE, Jenson HB, Stanton BF (2007). Nelson textbook of pediatrics.

[3] Chang LC, Haggstrom AN, Drolet BA, Baselga E, Chamlin SL, Garzon MC (2008). Growth characteristics of infantile hemangiomas: implications for management. Pediatrics.

[4] Bennett ML, Fleischer AB, Chamlin SL, Frieden IJ (2001). Oral corticosteroid use is effective for cutaneous hemangiomas: an evidence-based evaluation. Arch Dermatol.

[5] Price CJ, Lattouf C, Baum B, McLeod M, Schachner LA, Duarte AM (2011). Propranolol vs Corticosteroids for infantile Hemangiomas: a multicenter retrospective analysis. Arch Dermatol.

[6] Leaute-Labreze C, Dumas de la Roque E, Hubiche T, Boralevi F, Thambo JB, Taieb A (2008). Propranolol for severe hemangiomas of infancy. N Engl J Med.

[7] Chik KK, Luk CK, Chan HB, Tan HY (2010). Use of propranolol in infantile hemangioma Chinese children. Hoong Kong Med J.

[8] Sans V, Dumas de la Roque E, Berge J, Grenier N, Boralevi F, Mazereeuw-Hautier (2009). Propranolol for severe infantile hemangiomas: follow-up report. Pediatrics.

[9] Lawley LP, Siegfried E, Todd JL (2009). Propranolol treatment for hemangioma of infancy: risks and recommendations. Pediatr Dermatol.

[10] Koaya ACA, Choo MM, Nathan AM, Omar A, Lim CT (2011). Combined low dose oral propranolol and Oral Prednisolone as First-Line Treatment in Periocular Infantile Hemangiomas. J Ocular Pharmacology and Therapeutics.

[11] Frieden IJ, Haggstrom AN, Drolet BA, Mancini AJ, Friedlander SF (2005). Infantile hemangiomas: current knowledge, future directions. Proceedings of a Research Workshop on Infantile Hemangiomas 2005.

[12] Bertrand J, McCuaig C, Dubois J, Hatami A, Ondrejchak S, Powell J (2011). Propranolol versus prednisone in the treatment of infantile hemangiomas: a retrospective comparative study. Pediatr Dermatol.

[13] Buckmiller LM, Munson PD, Dyamenahalli U, Dai Y, Richter GT (2010). Propranolol for IH: early experience at a tertiary vascular anomalies center. Layngoscope.

